# 2,4,6-Trifluoro­aniline

**DOI:** 10.1107/S1600536808035083

**Published:** 2008-10-31

**Authors:** Richard Betz, Peter Klüfers

**Affiliations:** a Ludwig-Maximilians Universität, Department Chemie und Biochemie, Butenandtstrasse 5–13 (Haus D), 81377 München, Germany

## Abstract

The title compound, C_6_H_4_F_3_N, is a fluoro derivative of aniline. The mol­ecule shows non-crystallographic mirror symmetry. Bond lengths are normal. The C—C—C angles show some deviation from the expected ideal values by up to 5°, a finding which is in accordance with a similar structure in the literature. In the crystal structure H⋯F contacts and H⋯N contacts lead to the formation of sheets whose surfaces are made up by the hydro­phobic phenyl rings.

## Related literature

For the crystal structure of a related compound, see: Gdaniec (2007 ▶). For graph-set analysis, see: Bernstein *et al.* (1995 ▶); Etter *et al.* (1990 ▶).
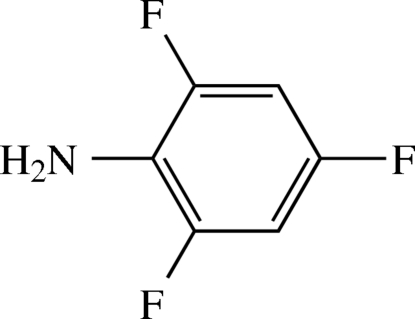


## Experimental

### 

#### Crystal data

C_6_H_4_F_3_N
*M* *_r_* = 147.10
Orthorhombic, 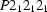

*a* = 6.3220 (6) Å
*b* = 24.792 (2) Å
*c* = 3.8545 (5) Å
*V* = 604.14 (11) Å^3^
*Z* = 4
Mo *K*α radiation
μ = 0.16 mm^−1^
*T* = 200 (2) K
0.50 × 0.09 × 0.05 mm

#### Data collection

Oxford Diffraction KappaCCD diffractometer
Absorption correction: multi-scan (SCALE3 ABSPACK in *CrysAlis RED*; Oxford Diffraction, 2005 ▶)) *T*_min_ = 0.921, *T*_max_ = 0.992
4517 measured reflections
775 independent reflections
489 reflections with *I* > 2σ(*I*)
*R*_int_ = 0.034

#### Refinement

*R*[*F* ^2^ > 2σ(*F* ^2^)] = 0.034
*wR*(*F* ^2^) = 0.082
*S* = 0.94
775 reflections
91 parameters
H-atom parameters constrained
Δρ_max_ = 0.13 e Å^−3^
Δρ_min_ = −0.16 e Å^−3^

### 

Data collection: *CrysAlis CCD* (Oxford Diffraction, 2005 ▶); cell refinement: *CrysAlis RED* (Oxford Diffraction, 2005 ▶); data reduction: *CrysAlis RED*; program(s) used to solve structure: *SHELXS97* (Sheldrick, 2008 ▶); program(s) used to refine structure: *SHELXL97* (Sheldrick, 2008 ▶); molecular graphics: *ORTEPIII* (Burnett & Johnson, 1996 ▶) and *Mercury* (Macrae *et al.*, 2006 ▶); software used to prepare material for publication: *SHELXL97*.

## Supplementary Material

Crystal structure: contains datablocks I, global. DOI: 10.1107/S1600536808035083/rn2051sup1.cif

Structure factors: contains datablocks I. DOI: 10.1107/S1600536808035083/rn2051Isup2.hkl

Additional supplementary materials:  crystallographic information; 3D view; checkCIF report

## Figures and Tables

**Table 1:** Hydrogen-bond geometry (Å, °)

*D*—H⋯*A*	*D*—H	H⋯*A*	*D*⋯*A*	*D*—H⋯*A*
N1—H71⋯N1^i^	0.90	2.26	3.110 (3)	157
N1—H72⋯F3^ii^	0.88	2.31	3.086 (2)	147

Symmetry codes: (i) 
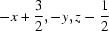
; (ii) 
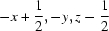
.

**Fig. 1.**
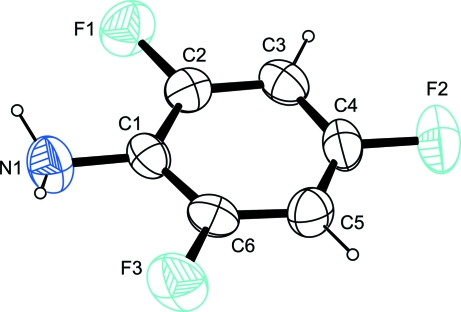
The molecular structure of the title compound, with atom labels and anisotropic displacement ellipsoids (drawn at 50% probability level) for non-H atoms.

**Fig. 2.**
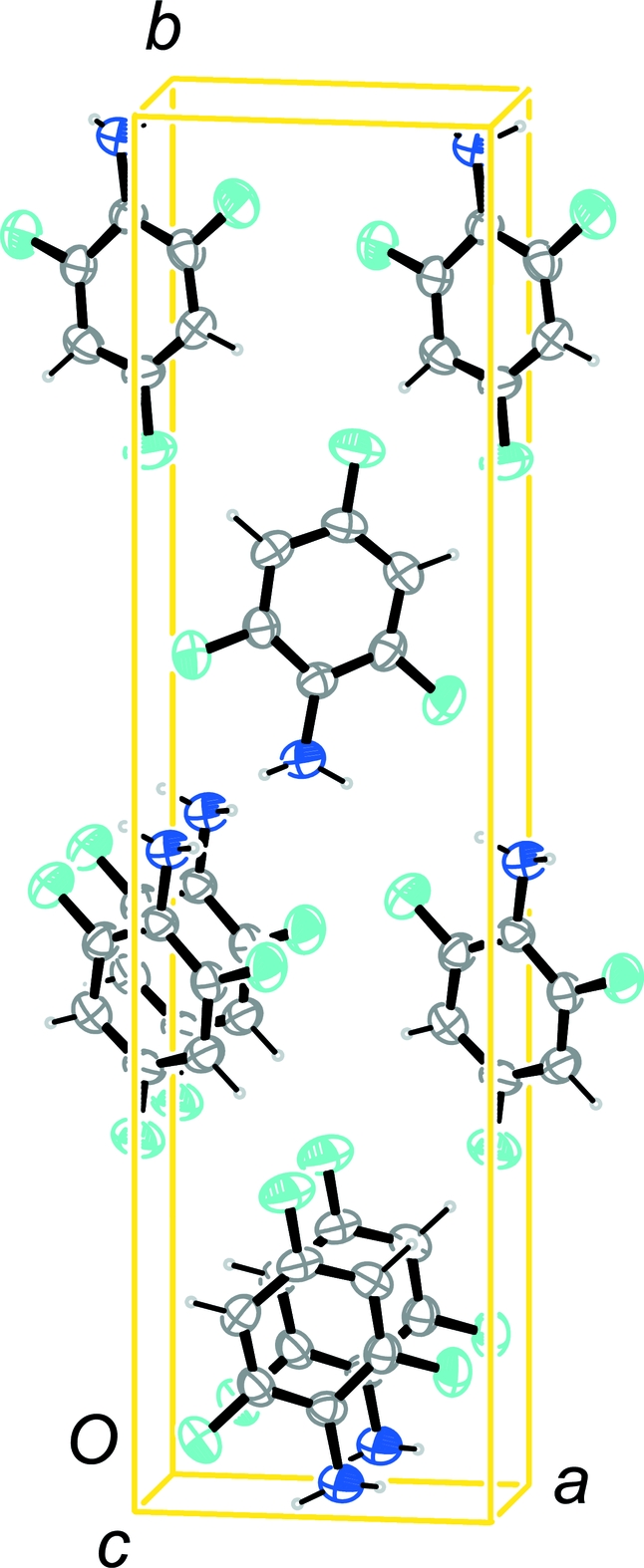
The packing of the title compound, viewed along [0 0 - 1].

**Fig. 3.**
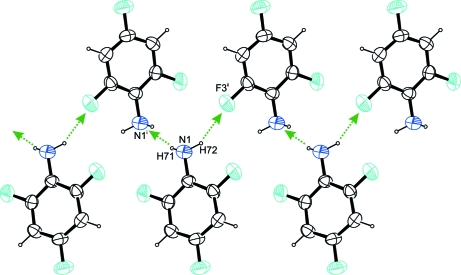
Intermolecular interactions in the crystal structure of the title compound, viewed along [0 0 1]. Symmetry operators: i -x + 3/2, -y, z - 1/2; ii -x + 1/2, -y, z - 1/2.

## supplementary crystallographic information

### Comment

In a program focused on the synthesis of derivatives of phenylarsonic acid a number of substituted aniline-derivatives were chosen as starting materials. In order to compare the influence of an arsonic group on the geometry of these starting materials, the crystal structure of 2,4,6-trifluoroaniline was elucidated by means of single-crystal X-ray diffraction.

In the molecule (Fig. 1) the C–C–C angles deviate from the expected ideal value of 120° by up to 5°. Angles bigger than the expected value are invariably found at C atoms bonded to fluorine, the smallest angle being present on the C atom bearing the amino group. This finding is in agreement with the situation observed in 2,3,4,5,6-pentafluoroaniline (Gdaniec, 2007).

In the crystal structure hydrogen bonds between fluorine and the amino group are present. If contacts whose ranges fall 0.2Å below the sum of van der Waals radii of the respective atoms are taken into consideration, only one of the F atoms in *ortho* position to the amino group is participating in these intermolecular interactions. These connect the molecules into sheets parallel to [1 0 1]. The surfaces of these sheets are made up by the aromatic moieties (Fig. 2 and Fig. 3). In terms of graph-set analysis (Etter *et al.*, 1990; Bernstein *et al.*, 1995) the N–H···F pattern should be assigned a C(5) descriptor on the unitary level while the remaining H atom on nitrogen participates in a cooperative chain of hydrogen bonds (N–H···N).

### Experimental

The compound was obtained commercially from Fluorochem. Crystals suitable for X-ray diffraction studies were obtained upon cooling the compound to 4 °C in a fridge.

### Refinement

All H atoms were located in a difference map and refined as riding on their parent atoms with *U*_iso_(H) values of 1.2 *U*_eq_(C) and 1.2 *U*_eq_(N).

Due to the absence of a significant anomalous scatterer in the molecule, the Flack parameter is meaningless. Friedel opposites were merged and the absolute structure parameter was removed from the CIF file.

### Crystal data

**Table:**

C_6_H_4_F_3_N	*F*(000) = 296
*M**_r_* = 147.10	*D*_x_ = 1.617 Mg m^−^^3^
Orthorhombic, *P*2_1_2_1_2_1_	Mo *K*α radiation, λ = 0.71073 Å
Hall symbol: P 2ac 2ab	Cell parameters from 1719 reflections
*a* = 6.3220 (6) Å	θ = 4.1–26.3°
*b* = 24.792 (2) Å	µ = 0.16 mm^−^^1^
*c* = 3.8545 (5) Å	*T* = 200 K
*V* = 604.14 (11) Å^3^	Rod, colourless
*Z* = 4	0.50 × 0.09 × 0.05 mm

### Data collection

**Table:**

Oxford Diffraction KappaCCD diffractometer	775 independent reflections
Radiation source: fine-focus sealed tube	489 reflections with *I* > 2σ(*I*)
graphite	*R*_int_ = 0.034
ω scans	θ_max_ = 26.4°, θ_min_ = 4.1°
Absorption correction: multi-scan (SCALE3 ABSPACK in CrysAlis RED; Oxford Diffraction, 2005))	*h* = −7→7
*T*_min_ = 0.921, *T*_max_ = 0.992	*k* = −30→30
4517 measured reflections	*l* = −4→3

### Refinement

**Table:**

Refinement on *F*^2^	Primary atom site location: structure-invariant direct methods
Least-squares matrix: full	Secondary atom site location: difference Fourier map
*R*[*F*^2^ > 2σ(*F*^2^)] = 0.034	Hydrogen site location: difference Fourier map
*wR*(*F*^2^) = 0.082	H-atom parameters constrained
*S* = 0.94	*w* = 1/[σ^2^(*F*_o_^2^) + (0.0505*P*)^2^] where *P* = (*F*_o_^2^ + 2*F*_c_^2^)/3
775 reflections	(Δ/σ)_max_ < 0.001
91 parameters	Δρ_max_ = 0.13 e Å^−^^3^
0 restraints	Δρ_min_ = −0.16 e Å^−^^3^

### Fractional atomic coordinates and isotropic or equivalent isotropic displacement parameters (Å^2^)

**Table:**

	*x*	*y*	*z*	*U*_iso_*/*U*_eq_	
F1	0.8818 (2)	0.10411 (5)	−0.1028 (5)	0.0618 (5)	
F2	0.4694 (2)	0.24248 (5)	0.3783 (5)	0.0712 (6)	
F3	0.2254 (2)	0.06360 (6)	0.3828 (5)	0.0715 (6)	
N1	0.5886 (3)	0.02701 (7)	0.0726 (7)	0.0564 (7)	
H71	0.6769	0.0202	−0.1054	0.068*	
H72	0.4647	0.0109	0.0632	0.068*	
C1	0.5551 (4)	0.08172 (8)	0.1384 (7)	0.0398 (6)	
C2	0.7034 (4)	0.12061 (9)	0.0624 (7)	0.0414 (6)	
C3	0.6805 (4)	0.17436 (8)	0.1376 (7)	0.0447 (7)	
H3	0.7868	0.2000	0.0811	0.054*	
C4	0.4963 (4)	0.18920 (8)	0.2983 (7)	0.0451 (7)	
C5	0.3394 (4)	0.15382 (8)	0.3828 (8)	0.0480 (7)	
H5	0.2124	0.1652	0.4922	0.058*	
C6	0.3757 (4)	0.10062 (9)	0.3004 (7)	0.0444 (7)	

### Atomic displacement parameters (Å^2^)

**Table:**

	*U*^11^	*U*^22^	*U*^33^	*U*^12^	*U*^13^	*U*^23^
F1	0.0434 (9)	0.0688 (9)	0.0731 (11)	0.0014 (7)	0.0104 (9)	−0.0040 (10)
F2	0.0870 (11)	0.0420 (8)	0.0845 (14)	0.0118 (8)	−0.0120 (14)	−0.0085 (10)
F3	0.0534 (9)	0.0641 (9)	0.0969 (14)	−0.0156 (7)	0.0105 (12)	0.0123 (10)
N1	0.0574 (14)	0.0408 (11)	0.0712 (18)	−0.0029 (10)	−0.0014 (15)	−0.0017 (12)
C1	0.0441 (13)	0.0354 (12)	0.0397 (16)	−0.0004 (10)	−0.0070 (14)	0.0045 (12)
C2	0.0379 (13)	0.0476 (14)	0.0386 (16)	0.0022 (11)	−0.0023 (13)	0.0011 (13)
C3	0.0503 (17)	0.0412 (14)	0.0426 (17)	−0.0103 (11)	−0.0064 (16)	0.0057 (14)
C4	0.0573 (16)	0.0326 (12)	0.0453 (17)	0.0063 (12)	−0.0101 (16)	−0.0018 (12)
C5	0.0445 (16)	0.0496 (14)	0.0499 (17)	0.0094 (12)	0.0004 (16)	0.0009 (16)
C6	0.0408 (13)	0.0473 (14)	0.0453 (19)	−0.0087 (12)	0.0001 (14)	0.0094 (13)

### Geometric parameters (Å, °)

**Table:**

F1—C2	1.358 (3)	C1—C6	1.377 (3)
F2—C4	1.367 (2)	C2—C3	1.371 (3)
F3—C6	1.359 (2)	C3—C4	1.369 (4)
N1—C1	1.396 (3)	C3—H3	0.9500
N1—H71	0.9009	C4—C5	1.364 (3)
N1—H72	0.8798	C5—C6	1.376 (3)
C1—C2	1.377 (3)	C5—H5	0.9500
			
C1—N1—H71	114.6	C2—C3—H3	121.7
C1—N1—H72	108.3	C5—C4—F2	118.5 (2)
H71—N1—H72	115.8	C5—C4—C3	123.6 (2)
C2—C1—C6	114.8 (2)	F2—C4—C3	117.9 (2)
C2—C1—N1	122.6 (2)	C4—C5—C6	116.1 (2)
C6—C1—N1	122.5 (2)	C4—C5—H5	121.9
F1—C2—C3	118.7 (2)	C6—C5—H5	121.9
F1—C2—C1	117.04 (19)	F3—C6—C5	118.5 (2)
C3—C2—C1	124.3 (2)	F3—C6—C1	116.9 (2)
C4—C3—C2	116.5 (2)	C5—C6—C1	124.6 (2)
C4—C3—H3	121.7		
			
C6—C1—C2—F1	−178.9 (2)	F2—C4—C5—C6	−179.2 (2)
N1—C1—C2—F1	4.2 (4)	C3—C4—C5—C6	0.7 (4)
C6—C1—C2—C3	0.0 (4)	C4—C5—C6—F3	179.2 (3)
N1—C1—C2—C3	−176.9 (3)	C4—C5—C6—C1	−0.8 (4)
F1—C2—C3—C4	178.8 (2)	C2—C1—C6—F3	−179.5 (2)
C1—C2—C3—C4	−0.1 (4)	N1—C1—C6—F3	−2.7 (4)
C2—C3—C4—C5	−0.2 (4)	C2—C1—C6—C5	0.5 (4)
C2—C3—C4—F2	179.6 (2)	N1—C1—C6—C5	177.3 (3)

### Hydrogen-bond geometry (Å, °)

**Table:**

*D*—H···*A*	*D*—H	H···*A*	*D*···*A*	*D*—H···*A*
N1—H71···N1^i^	0.90	2.26	3.110 (3)	157
N1—H72···F3^ii^	0.88	2.31	3.086 (2)	147

Symmetry codes: (i) −*x*+3/2, −*y*, *z*−1/2; (ii) −*x*+1/2, −*y*, *z*−1/2.
